# Commissioning and acceptance testing of a CyberKnife linear accelerator

**DOI:** 10.1120/jacmp.v8i3.2473

**Published:** 2007-08-08

**Authors:** Subhash C. Sharma, Joseph T. Ott, Jamone B. Williams, Danny Dickow

**Affiliations:** ^1^ Parkview Comprehensive Cancer Center Fort Wayne Indiana U.S.A.

**Keywords:** Cyber Knife, commissioning, radiosurgery

## Abstract

Acceptance testing and commissioning of a CyberKnife robotic stereotactic radiosurgery system was performed in April 2006. The CyberKnife linear accelerator produces a photon beam of 6 MV nominal energy, without the use of a flattening filter. Clinically measured tissue–phantom ratios, off‐center ratios, and output factors are presented and compared with similar data from other CyberKnife sites throughout the United States. In general, these values agreed to within 2%.

PACS number: 87.53.Dq

## I. INTRODUCTION

A CyberKnife G3 robotic stereotactic radiosurgery system (Accuray, Sunnyvale, CA), was installed at the Parkview Comprehensive Cancer Center, Fort Wayne, Indiana, in April 2006. CyberKnife uses frameless real‐time image guidance technology and computer‐controlled robotics to treat tumors anywhere in the body with submillimeter accuracy. In addition to intracranial tumors, which can be treated by many radiosurgical modalities, tumors in the spine, lung, liver, prostate, and pancreas are currently being treated using the CyberKnife system.^(^ ^1^ ^,^ ^2^ ^)^ The CyberKnife image guidance allows for continuous tracking and correction of tumor and patient movement, in real time, throughout treatment delivery.

The CyberKnife linear accelerator (LINAC) produces a photon beam of 6 MV nominal energy. Because CyberKnife is a dedicated, single‐energy radiosurgery system, the treatment beam is accomplished without the aid of a bending magnet or flattening filter. The compact, lightweight LINAC head is attached to the end of a robotic arm that is free to rotate and translate with 6 degrees of freedom. As a result, treatments are non‐isocentric, allowing for unique beam angles and orientations. Treatment field sizes are determined by the use of interchangeable secondary circular cones of diameters 5.0, 7.5, 10.0, 12.5, 15.0, 20.0, 25.0, 30.0, 35.0, 40.0, 50.0, and 60.0 mm.

The Parkview CyberKnife was put through acceptance testing and commissioning, including absolute dose calibration, using the American Association of Physicists in Medicine (AAPM) TG‐51 protocol.^(3)^ Clinical dosimetry measurements such as tissue‐phantom ratios (TPRs), off‐center ratios (OCRs), and secondary collimator output factors are presented here. In addition, these measurements are compared to a set of nationwide average values.

## II. MATERIALS AND METHODS

All clinical dosimetry data were acquired using

- a computer‐controlled measuring system (MP3‐M Therapy Beam Analyzer: PTW, Freiburg, Germany),
- a Unidos electrometer (PTW), and
- a PTW 60008 photon diode (PTW).

The absolute dose calibration of the accelerator output was accomplished using

- a TN30013 ion chamber (PTW) and
- a Unidos electrometer.

## III. RESULTS

### A. Clinical dosimetry data

#### *A.1 Central‐axis TPR*

The TPR measurements were carried out using the photon diode positioned in the computer‐controlled water phantom. Central axis TPRs were measured for each secondary collimator at depths ranging from 0 cm to 29.9 cm (Table 1). The reference depth used for normalization of the TPR data was 1.5 cm for all collimator sizes, which is the nominal depth of maximum dose $\left(d_{\text{max}}\right)$. All measurements were made at a source–detector distance (SDD) of 80 cm.

**Table 1:** CyberKnife^a^ central‐axis measurements of tissue–phantom ratios for selected depths, using a source–axis distance of 80 cm

						Collimator (mm)						
Depth (cm)	5	7.5	10	12.5	15	20	25	30	35	40	50	60
0	0.820	0.736	0.711	0.697	0.673	0.665	0.665	0.663	0.664	0.667	0.669	0.669
1	1.015	1.003	0.996	0.988	0.982	0.976	0.974	0.978	0.976	0.976	0.973	0.972
1.5	1.000	1.000	1.000	1.000	1.000	1.000	1.000	1.000	1.000	1.000	1.000	1.000
2	0.975	0.979	0.982	0.985	0.987	0.991	0.994	0.992	0.994	0.994	0.996	0.996
3	0.925	0.931	0.936	0.942	0.945	0.953	0.959	0.957	0.961	0.963	0.968	0.969
5	0.826	0.833	0.839	0.845	0.850	0.860	0.868	0.872	0.877	0.883	0.891	0.896
7	0.741	0.749	0.757	0.764	0.769	0.780	0.789	0.793	0.800	0.807	0.818	0.825
10	0.630	0.639	0.647	0.654	0.660	0.671	0.679	0.684	0.691	0.700	0.712	0.722
14	0.510	0.520	0.528	0.535	0.540	0.550	0.557	0.563	0.569	0.578	0.590	0.599
18	0.415	0.426	0.434	0.440	0.445	0.454	0.460	0.464	0.471	0.478	0.489	0.498
25	0.294	0.303	0.310	0.316	0.320	0.326	0.332	0.335	0.340	0.345	0.354	0.362
30	0.233	0.241	0.247	0.252	0.256	0.261	0.265	0.269	0.272	0.277	0.284	0.290

a Accuray, Sunnyvale, CA.

#### *A.2 OCR*

**Figure 1:**
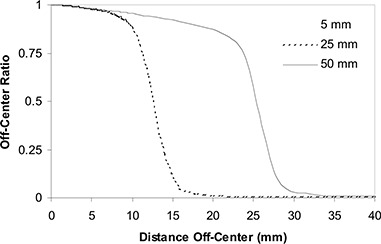
Representative off‐center ratios for the CyberKnife (Accuray, Sunnyvale, CA): 80 cm source–axis distance, 1.5 cm depth. Only 5‐, 25‐, and 50‐mm cone off‐center ratios are shown.

The OCR at a particular depth is the ratio of the absorbed dose at a given off‐axis point relative to the dose at central axis. Measurements of OCR were carried out by conducting orthogonal scans across the field at a variety of depths. Individual OCR values were calculated by averaging each side of the crossplane and inplane scans; therefore, each entry in the OCR data table is the average of four measured values. For each secondary collimator, OCR measurements were made for depths ranging from 1.5 cm to 25 cm and off‐axis distances ranging from 0 cm to 4.9 cm. Fig. 1 shows the measurements of OCR taken at $d_{\max}$ (1.5 cm), for the 5‐, 25‐, and 50‐mm cones.

#### *A.3 Output factors*

The output factor is a field size–dependent correction for the output of the CyberKnife accelerator. It is defined as the ratio of absorbed dose of a particular field size relative to the dose at a reference field size. Field size is determined by choice of collimator size and SDD. Similar to conventional LINAC photon beams, the denoted field size for a given collimator—for example, 60 mm—is defined dosimetrically. The measured field size is the area encompassed by 50% of the central‐axis maximum dose, at a reference SDD of 80 cm. In the measurements reported here, the reference field size was 60 mm and the depth of measurement was $d_{\max}$. Table 2 presents the measured output factors for each cone.

**Table 2:** CyberKnife^a^ output factors normalized to the 60‐mm collimator, using a source–axis distance of 80 cm, with $d_{\max}$ 1.5 cm

Collimator (mm)	Output factor
5.0	0.704
7.5	0.877
10.0	0.916
12.5	0.946
15.0	0.961
20.0	0.976
25.0	0.983
30.0	0.986
35.0	0.990
40.0	0.992
50.0	0.996
60.0	1.000

a Accuray, Sunnyvale, CA.

#### *A.4 Hand‐calculation verifications*

The current CyberKnife treatment planning system (CYRIS Multiplan 1.5.1) uses a correction‐based dose calculation algorithm.^(4)^ As a result, hand‐calculation verifications of treatment beams, using the foregoing clinical dosimetry data, are generally within a few percent. The output in centigrays per monitor unit (MU) for each clinical beam can be calculated using the equation

(1) D(cGyMU)=1(cGyMU)*OCR(coll,oad,deff)*(800SDD)2*TPR(rd,deff)*OF(rd),

where 1 cGy/MU is the calibrated output of the LINAC under reference conditions, and *coll* refers to the collimator size, *oad* to the off‐axis distance, $d_{\text{eff}}$ to the effective depth, and $r_{d}$ to the distance‐corrected field size. The squared term represents an inverse square correction on the output, relative to the reference distance of 80 cm. An independent MU calculation program (RadCalc, version 5.1: Lifeline Software, Tyler, TX) was used as a second check of the hand‐calculation verifications. It should be noted that future versions of Multiplan will use a dose calculation algorithm that treats the output factor as a function of both $r_{d}$ and SDD.

### B. Dose calibration and periodic output checks

Absolute dose calibration of the CyberKnife was accomplished in accord with the AAPM TG‐51 protocol. The CyberKnife output was calibrated to output 1 cGy per MU under reference conditions: 60 mm collimator, 80 cm source–axis distance (SAD), and 1.5 cm depth in water. Under the TG‐51 protocol, absolute calibration requires the use of a beam‐quality conversion factor, $k_{Q}$, which must be determined using a percent depth dose measurement.^(3)^ The parameters for this beam quality–defining percent depth dose measurement are very specific: 100 cm source‐to‐surface distance (SSD), 10×10 cm field size (at 100 cm), and depth of 10 cm. Therefore, an approximation must be made to accommodate the reference conditions of the CyberKnife.

The procedure that follows was used to obtain $k_{Q}$ for the CyberKnife LINAC beam. A 10‐cm percent depth dose was measured at an extended SSD of 100 cm using the 60‐mm collimator. The extended circular field size was converted to its equivalent square field. In this case, the 60‐mm collimator is equivalent to a square field of dimension 6.75 cm.^(4)^ A standard reference table was used as a benchmark for the percent depth dose measurement. The approximate percent depth dose for the CyberKnife with a 10×10 cm field size can be inferred from the reference table. Once the percent depth dose for the equivalent 10×10 cm field is obtained, the approximate value for $k_{Q}$ can be determined from the tables contained in the TG‐51 protocol.

The CyberKnife uses vented ion chambers, and therefore changes in environmental conditions can lead to variations in accelerator output. Daily output corrections are facilitated by performing an in‐air measurement before the first treatment patient of the day.

Each CyberKnife unit comes equipped with a “birdcage” attachment that can be fastened to the collimator assembly. The “birdcage” is a frame that holds the ion chamber, with buildup cap, at a reproducible position in air. In addition, the birdcage assembly is much more convenient for daily setup than is the scanning water tank or a Solid Water (Gammex rmi, Middleton, WI) phantom.

The output constancy factor is defined by the ratio

(2) CF=DTG−51RBirdcage(cGy/MUnC) ,

where $R_{\text{Birdcage}}$ is the temperature‐ and pressure‐corrected ion chamber reading, in nanocoulombs (nC), corresponding to the in‐air birdcage measurement, and $D_{\text{TG}-51}$ is the actual calibrated output (cGy/MU) of the accelerator, which will have a nominal value of 1.0 cGy/MU. The daily output correction factor is obtained by applying the output constancy factor to the daily temperature‐ and pressure‐corrected birdcage measurement, and then, if necessary, scaling the result to obtain 1 cGy/MU.

### C. End‐to‐end test

One periodic quality assurance technique unique to the CyberKnife is the end‐to‐end test. The end‐to‐end test is a quantitative measure of the CyberKnife system's overall targeting accuracy. It integrates patient setup, computed tomography acquisition accuracy, treatment planning, robot movement, image processing, beam alignment, and treatment delivery—all essential steps of any patient treatment.

The end‐to‐end test uses an anthropomorphic head phantom to simulate an actual patient treatment. Orthogonal radiochromic films can be placed within the phantom, which allows for subsequent comparison of planned and delivered dose distributions. Following irradiation, a proprietary film analysis software package provided by Accuray calculates the centroid of the delivered dose distribution. The distance between the centers of the planned and delivered dose distributions is a measure of the system's submillimeter accuracy.

Each end‐to‐end test that we have conducted has resulted in deviations of less than 1 mm between the planned and delivered dose distributions. These results reflect the accuracy and stability of the system's non‐isocentric beam geometry. Radiochromic film is suitable for this process because of its relative insensitivity to ambient light and also because of the relatively high dose characteristics of radiosurgery procedures.

## IV. DISCUSSION

The measured TPRs and output factors were found to be in excellent agreement with average multi‐site data, which are maintained and provided by Accuray. The multi‐site data are an average of the measurements made by several CyberKnife sites throughout the United States. In most cases, each of our measurements was within ±2% of the average data. These findings are reassuring, considering the small field sizes used in the measurements. Tables 3 and 4 present comparisons between the measured data (TPRs and output factors) and the average data.

The greatest discrepancy between the measured data and the average data occurred for the surface measurement of the TPR. These results are probably attributable in large part to the different measuring devices in use at the respective CyberKnife sites. In addition to a dependence on slight variations in the actual point of measurement, surface measurements are highly dependent on the type and size of the measuring instrument.^(5)^

Recent studies have shown that the photon diode used in the present measurements (PTW 60008) tends to over‐respond during small‐field measurements,^(^ ^6^ ^,^ ^7^ ^)^ a result of increased electron scattering from the metallic shielding that partially encapsulates the active volume of the diode. It is interesting to note that, when compared with Monte Carlo calculations, our measurements, particularly the output factors for the smaller cones, agree very well with the average multi‐site data—despite the over‐response.^(6)^

The measured TPRs for the 60‐mm collimator were also compared to the 6‐MV tissue‐maximum ratios described by Day and Aird.^(4)^ As can be seen in Table 5, the discrepancies between our measured values and those by Day and Aird increase with depth. One reason may be the presence of a flattening filter in conventional LINACs, which would lead to a harder photon beam.

**Table 3:** Percentage disagreement between the measured tissue–phantom ratios from Parkview Health^a^ and standard tissue–phantom ratios provided by Accuray^b^

						Collimator (mm)^c^						
Depth (cm)	5	7.5	10	12.5	15	20	25	30	35	40	50	60
0	27.04	20.99	20.15	21.56	19.13	19.63	19.72	19.20	19.49	18.96	19.20	18.25
1	0.54	0.44	0.61	0.59	0.62	0.65	0.68	1.21	0.92	1.01	0.84	0.50
1.5	0.00	0.00	0.00	0.00	0.00	0.00	0.00	0.00	0.00	0.00	0.00	0.00
2	−0.06	−0.08	−0.02	−0.14	−0.29	−0.30	−0.18	−0.41	−0.36	−0.37	−0.35	−0.41
3	−0.04	−0.16	0.01	0.15	−0.05	−0.04	0.15	−0.54	−0.38	−0.32	−0.21	−0.42
5	−0.30	−0.70	−0.63	−0.54	−0.64	−0.62	−0.50	−0.79	−0.88	−0.59	−0.66	−0.78
7	−0.43	−0.70	−0.36	−0.25	−0.50	−0.43	−0.27	−0.71	−0.75	−0.46	−0.60	−0.78
10	−0.63	−0.96	−0.86	−0.69	−0.72	−0.66	−0.67	−0.99	−1.03	−0.54	−0.70	−0.86
14	−0.62	−1.07	−1.07	−0.91	−0.89	−0.95	−0.84	−1.08	−1.12	−0.80	−0.88	−1.13
18	−0.67	−1.12	−1.31	−1.08	−1.09	−1.16	−1.00	−1.44	−1.42	−0.65	−1.14	−1.48
25	−0.09	−0.94	−1.62	−1.58	−1.73	−1.83	−1.50	−1.92	−1.81	−1.54	−1.38	−1.75
30	0.18	−0.60	−1.78	−1.70	−1.85	−1.65	−1.73	−1.83	−1.80	1.18	−1.33	−1.97

a Fort Wayne, IN.

b Sunnyvale, CA.

c Negative values represent instances in which the standard tissue–phantom ratio is greater than the measured ratio.

**Table 4:** Percentage disagreement between measured output factors from Parkview Health^a^ and standard output factors provided by Accuray^b^

Collimator (mm)	Parkview	Accuray average	% Difference
5	0.704	0.707	−0.42
7.5	0.877	0.861	1.86
10	0.916	0.905	1.22
12.5	0.946	0.937	0.96
15	0.961	0.954	0.73
20	0.976	0.972	0.41
25	0.983	0.979	0.41
30	0.986	0.983	0.31
35	0.990	0.987	0.30
40	0.992	0.990	0.20
50	0.996	0.995	0.10
60	1.000	1.000	0.00

a Fort Wayne, IN.

b Sunnyvale, CA.

**Table 5:** Comparison between tissue–phantom ratios measured with the 60‐mm collimator and tissue‐maximum ratios reported by Day and Aird,^(4)^ using a field size of 5.4×5.4 cm

Depth (cm)	Day and Aird	Parkview Health^a^	% Difference
1.5	1.0	1.0	0
3	0.973	0.969	0.45
4	0.944	0.932	1.31
5	0.912	0.896	1.79
10	0.749	0.722	3.74
15	0.607	0.572	6.19
20	0.488	0.455	7.25
25	0.393	0.362	8.56
30	0.317	0.290	9.17

a Fort Wayne, IN.

## ACKNOWLEDGMENTS

The authors thank Larry Thompson and Bill Main of Accuray, Inc., for reviewing this manuscript before publication and for providing valuable data for comparison.
